# The Institutional Library

**Published:** 1913-05-17

**Authors:** 


### THE INSTITUTIONAL LIBRARY.

The Medical Annual, 1913. (Bristol: J. Wright and Co., Ltd. Pp. 702. Price 8s. 6d. net.)

This admirable annual continues on that even career of assured success to which the editor and publisher have long been accustomed. Good as the thirty predecessors of this year's volume have been, we do not think they contain a better summary of the past year's work and progress than is here published. May the monotony of unbroken prosperity long attend the "Medical Annual"; while it is conducted on the lines of 1913 and preceding years, we are perfectly certain the wish will be fulfilled.

Early English Water-Colour. By C. E. Hughes. (London: Methuen. 1913. Pp. 195. Price 2s. 6d. net.)

In this well arranged and highly presentable volume the author gives a scholarly account of the water-colour school of English painters whose crowning glory was J. M. W. Turner. As arbitrary limits of this epoch, Mr. Hughes excludes all painters born before 1720 and after 1820; and he takes the death of Turner (1851) as the dividing line between the old school and the new. Anyone interested in the art of Constable, Cox, Varley, Bonington, and many others of a hundred years or so ago will find here due appreciation without indiscriminate worship or blind adulation.

Gynæcology for Nurses and Gynæcological Nursing. By Comyns Berkeley, M.D., F.R.C.P. (London: The Scientific Press. Second Edition. 1913. Pp. 166. Price 2s. 6d. net.)

As was to be expected, the first edition of this excellent little handbook attained a rapid popularity. In this new edition careful revision has still further improved it and increased its utility. We do not doubt that it will easily maintain the position it already holds in the estimation of those for whom it is written; because it is so thoroughly practical in spirit and letter, and so obviously the work of an experienced teacher who knows exactly what the difficulties of the subject are for those in pupilage, and how they may best be surmounted.

Medical Electricity. By H. Lewis Jones, M.A., M.D. (London: H. K. Lewis. Sixth Edition. 1913. Pp. 551. Price 12s. 6d. net.)

The appearance of the sixth edition of this well-known standard text-book has been signalised by an extensive revision of the contents, which shows that the author is determined to keep the position he has achieved for his book. This entails incessant effort, for progress is so rapid and novelties so frequent in this young science of electro-therapeutics that much vigilance is required to keep abreast of the times. Suffice it that in this book the effort is adequately and successfully made, and that the old qualities of thoroughness and dependability are just as much in evidence as in the previous editions.

Maternity Nursing. By Sarah Macdonald. (London: Methuen. 1913. Price 3s. 6d. net.)

This book is written for "monthly nurses" as opposed to the more highly instructed "midwives" who seek to fill the requirements of the Central Midwives Board. We are not sure, for our own part, that it is really wise to create a distinction between these two varieties of maternity nurse. A qualified midwife makes the best monthly nurse; but a less educated and therefore less expensive assistant is a public want, so from the point of view of expediency there is something to be said in her favour. The present volume presents an outline of her duties which is quite sound and intelligible. Apparently the practitioners of the Salford neighbourhood do not wear gloves when examining parturient women; but as those of other localities often do, it would be well to mention the fact in the chapter dealing with the preparation of lotions, etc., for this purpose. The last (sixth) lecture is less satisfactory than the others. The authoress professes to tell her pupils plain truths about abortion and similar topics; but instead of doing so she wraps up her instructions in such vague generalities that she might just as well have left the subject untouched. Otherwise we have nothing but commendation for a really sensible book.

Pharmacy and Materia Medica for Nurses. By Horace Finnemore, B.Sc., F.I.C. (London: H. H. G. Grattan. 1913. Pp. 237. Price 2s. 6d.)

A cordial welcome should be accorded to Mr. Finnemore's effort to place before the nursing profession a reliable and practical text-book of pharmacy and materia medica. The nurse who really cares about her profession, and is animated with a desire to know all she can of what is legitimately within her own sphere, will find a great deal of very helpful instruction in this book. The mere smattering which is so dangerous is here carefully avoided, and sufficient details are given of drugs and methods to make the nurse's knowledge of practical value. In Part I. the methods of pharmacy and pharmaceutical chemistry are described, followed by the descriptions of all the generally used drugs classified according to their chief functions. Part II. consists of chapters on prescriptions, weights and measures, dispensing and prescribing abbreviations. This portion of the book is well done, and should prove a useful exposition of a somewhat difficult subject for the average nurse. We note that in describing the signs used in prescriptions, Mr. Finnemore refers to the fact that the sign for "one" is often written j instead of i, but does not offer any reason therefor; is it not to show that the j is the final one of a series, as in viij or vj, and to obviate ambiguity in careless writing? Among the excellent lists of abbreviations we find few omissions, but certainly the suffix *que* should be included. A useful section is that on the making of lotions of definite strengths and on the comparative cost of some antiseptic solutions. Among the lists there are a few slips left uncorrected, such as *mangani* for *manganesii*, and on page 178 is an error in a prescription.

